# Postpartum Complications Modified TBL

**DOI:** 10.5070/M5.52264

**Published:** 2026-04-30

**Authors:** Lauren E Lamparter

**Affiliations:** University of California, Irvine, Department of Emergency Medicine, Orange, CA

## Abstract

**Audience:**

The intended audience for this modified TBL are resident physicians of all years, PGY1-4.

**Introduction:**

Biological females represent 50% of the population we encounter in the emergency department (ED), and following delivery, the ED is often the main resource for postpartum patients to seek urgent/emergent care. The US is one of the most dangerous developed nations for pregnant women, with about 1/3 of pregnancy related deaths occurring in the postpartum period.^1^,^2^ It is presumed that many of these deaths are related to lack of support or insurance for close follow up after delivery.^2^ The three most common reasons for postpartum hospital readmission and mortality are cardiovascular related disorders (such as hypertension, eclampsia, and cardiomyopathy), hemorrhage, and infections.^2^,^3^ While most common, those are not exhaustive, and postpartum women are at risk for many other complications, such as breastfeeding related problems, which will cause them to present to the ED.

As the front lines for patients with limited support or access to medical care, emergency physicians (EPs) need to understand how to recognize and intervene on the common complications faced by postpartum women. It is paramount that resident physicians have adequate training to consider and treat the unique medical concerns of this vulnerable population. This team-based learning (TBL) activity will prepare resident EPs to recognize and manage common postpartum complications.

**Educational Objectives:**

By the end of this session learners will be able to: 1) identify the timeline in which postpartum complications typically occur, 2) discuss the specific differential diagnoses common in the postpartum period, 3) recognize risk factors for endometritis and mastitis, 4) recognize the presentation of postpartum cardiomyopathy and pituitary infarction, 5) formulate a treatment plan for postpartum endometritis including appropriate antibiotics and disposition, 6) formulate a treatment plan for mastitis including appropriate antibiotics and disposition, 7) formulate an appropriate assessment plan for pituitary infarction with appropriate disposition, and 8) formulate an appropriate treatment plan and disposition for postpartum cardiomyopathy.

**Educational Methods:**

This is a modified team-based learning (mTBL) activity in which learners do not have learner responsible content (LRC) prior to the educational session. The multiple-choice cases can be used as an individual readiness assessment test (iRAT) followed by group discussion in a group readiness assessment test (gRAT), or without an iRAT and completed in small groups as a gRAT followed by instructor feedback and summary. Following the gRAT, there is a group application exercise (GAE) with summary cases.

**Research Methods:**

This mTBL was evaluated by learners immediately following the educational session using a post-participation survey. A Likert scale was used to assess the learner’s perception of the effectiveness of this educational format, relevance of the content to practice as EPs, and learner engagement with the mTBL.

**Results:**

The post mTBL survey had a response rate of 82% with 29/35 participants completing the evaluation. Overall, the learners rated the mTBL highly with an average total score of 4.75/5 on the Likert scale. Seventy-nine percent of learners strongly agreed (5/5 on the Likert scale) that the mTBL was effective and valuable compared to other educational activities and engaged their attention. Eighty-three percent of the learners strongly agreed (5/5 on the Likert scale) that the content was relevant to their practice of emergency medicine (EM).

**Discussion:**

Postpartum patients are a high risk, vulnerable population who present to the ED with a wide range of concerns. It is paramount that resident physicians are prepared to medically advocate for and treat these patients appropriately. This mTBL allowed the learners to practice engaging with and discuss the complex medical conditions faced by these patients to better serve them on their next ED shift. Learners found this content very relevant and directly relatable to their daily practice.

**Topics:**

Postpartum, eclampsia, endometritis, postpartum cardiomyopathy, breastfeeding, mastitis.

## USER GUIDE

**Table:** 

List of Resources:	
Abstract	1
User Guide	3
Learner Materials	5
iRAT	5
gRAT	6
GAE	10
Instructor Materials	12
RAT Key	13
GAE Key	17

**Learner Audience:**

This mTBL is appropriate for interns, and junior and senior residents.

**Time Required for Implementation:**

The instructor will need 30 min - 1 hour to review the provided materials and prepare to facilitate the mTBL. There is no LRC for this mTBL, though if desired, the instructor pre-reading could be used as LRC.

During the session, this TBL gRAT and GAE will be completed in 1 hour:

- 20 minutes allotted for the gRAT in small groups
- 5–10 minutes of instructor discussion of the gRAT
- 20 minutes for GAE
- 5 minutes of instructor discussion of the GAE
- It is recommended to send out the answer key to the gRAT and GAE following the session for independent learner review

**Recommended Number of Learners per Instructor:**

Learners should be broken up into independent groups of 4–5 residents. If able, add an attending facilitator to each group, supplied with an answer key to aid learner discussion. If unable to utilize attending facilitators, allot more time to the instructor discussion following small group discussion of the gRAT and GAE as a large group.

**Topics:**

Postpartum, eclampsia, endometritis, postpartum cardiomyopathy, breastfeeding, mastitis.

**Objectives:**

By the end of this session learners will be able to:

1. Identify the timeline in which postpartum complications typically occur.
2. Discuss the specific differential diagnoses common in the postpartum period.
3. Recognize risk factors for endometritis and mastitis.
4. Recognize the presentation of postpartum cardiomyopathy and pituitary infarction.
5. Formulate an appropriate treatment plan for postpartum endometritis with appropriate antibiotics and disposition.
6. Formulate an appropriate treatment plan for mastitis with appropriate antibiotics and disposition.
7. Formulate an appropriate treatment plan for pituitary infarction with appropriate disposition.
8. Formulate an appropriate treatment plan and disposition for postpartum cardiomyopathy.

### Linked objectives and methods

This is a modified team-based learning (mTBL) activity in which learners do not have LRC prior to the educational session. This format allows learners to participate in cooperative learning by engaging with others of different training length to assess, treat, and disposition complex postpartum patients. The multiple-choice cases can be used as an individual readiness assessment test (iRAT) followed by group discussion in a group readiness assessment test (gRAT), or without an iRAT and completed in small groups as a gRAT followed by instructor feedback and summary. Within the gRAT, learners will discuss the differential diagnoses, risk factors, and presentations of common postpartum complications (Objectives 1–4), and cases will be used for practice in appropriately treating and dispositioning these patients (Objectives 5–8). This is followed by a group application exercise (GAE) with summary cases that will reiterate the timeline of postpartum complications and allow learners to once more practice assessing and managing these high-risk patients.

### Recommended pre-reading for instructor

In addition to reviewing the material included in the TBL, the following background reading is recommended. *This reading could also be assigned asynchronously to the participating residents prior to completion of the TBL.*

- Rajpal M, Milliner B. Postpartum (within 1st month) emergencies and their management. emDocs. Published June 7, 2016. https://www.emdocs.net/postpartum-within-1st-month-emergencies-and-their-management/
- Tintinalli JE. Section 11: Obstetrics and gynecology. Chapter 100, postpartum endometritis, peripartum cardiomyopathy. *Tintinalli’s Emergency Medicine: A Comprehensive Study Guide*. 9th ed. McGraw-Hill Education; 2020:636.
- Tintinalli JE. Section 11: Obstetrics and gynecology. Chapter 104, breast disorders. *Tintinalli’s Emergency Medicine: A Comprehensive Study Guide*. 9th ed. McGraw-Hill Education; 2020:650–660.
- Tintinalli JE. Section 14: Chapter 165, headache. *Tintinalli’s Emergency Medicine: A Comprehensive Study Guide*. 9th ed. McGraw-Hill Education; 2020:1113.

### Results and tips for successful implementation

This mTBL was implemented virtually in Fall 2024. The participants included emergency medicine residents post grad years 1–3 and consisted of about 4–5 learners per group (with a mixed representation of post grad years) and 1–2 attending facilitators. A total of 35 learners and facilitators participated in the mTBL. Google forms were used for the gRAT and GAE with the learners manually entering their answers into the form while sharing a screen with the group. This mTBL can easily be adapted to an in-person format if the facilitator prints out the gRAT and GAE in preparation for the session, providing handouts for each group.

After implementation of this mTBL, a Likert scale survey was provided to all participants (both learners and facilitators) with a response rate of 82% with 29/35 participants completing the evaluation. Overall, the learners rated the mTBL highly with an average total score of 4.75/5 on the Likert scale. Seventy-nine percent of participants strongly agreed (5/5 on the Likert scale) that the mTBL was effective and valuable compared to other educational activities and engaged their attention. Eighty-three percent of participants strongly agreed (5/5 on the Likert scale) that the content was relevant to their practice of EM.

Written feedback was positive and supported continued use of the small group discussion format. Constructive feedback requested the addition of visual stimuli which could be added at the facilitator’s discretion, perhaps in the summary comments following the gRAT or GAE. For future implementation of this TBL, the instructor could also create a summary PowerPoint when reviewing the content, rather than verbally discussing the question answers with participants. However, most learners stated the verbal discussion was sufficient. It is recommended to send out the answers to the gRAT and GAE after session completion as a post-session review.

Limitations of this mTBL include the small sample size from implementation at one residency program and the potential bias of asking resident physicians to evaluate their own educational activity which could cause them to rate it more positively. Overall, however, this mTBL allowed the learners to practice engaging with and discussing the complex medical conditions faced by postpartum patients, allowing them to implement the assessment and treatment of these high-risk patients on their next shift.

## LEARNER MATERIALS

### Postpartum Complications mTBL: individual Readiness Assessment Test (iRAT)

In this modified TBL, the gRAT could be used as an iRAT to test individual readiness, if desired.

### Postpartum Complications mTBL: group Readiness Assessment Test (gRAT)

#### Section 1) General Postpartum Information

Complications unique to the postpartum period typically begin about 48 hours after delivery. For how long after the 48-hour period are patients at increased risk of postpartum complications? ________________________________________________________________

List the possible postpartum complications patients may present to the emergency department with: ______________________________________________________________

We commonly think about postpartum hemorrhage immediately following delivery, with the most common cause being uterine atony. However, patients can present with **late** postpartum hemorrhage, even weeks following delivery. What is the differential diagnosis for late postpartum hemorrhage? _______________________________________________________

A systolic blood pressure (BP) > ______ and a diastolic BP > _____ are concerning for a significant intrapartum or postpartum abnormality. Elevated blood pressure should cause you to consider ________________ which can develop acutely in the postpartum period. Pay careful attention to the BP of all young women in the event they are recently postpartum and did not say such in their initial triage assessment.

It is important to be aware of postpartum hemorrhage and postpartum preeclampsia as key contributors to morbidity and mortality in our pregnant patients. These topics are vast and need their own dedicated consideration, so we will not be covering them further today.

#### Section 2) Postpartum Infections

As with any procedure or surgery, pregnant patients are at risk for infections following delivery.

______________________ is the name of the uterine infection occurring postpartum, and you should assume this diagnosis, until proven otherwise, in any postpartum patient with a fever.

What are the risk factors for acquiring this uterine infection? ________________________ __________________________________________________________________________ Of these risk factors, _______________________________ is the most significant risk factor.

What are the most common bacteria causing uterine infections in the postpartum period? _____________________________________________________________________________

What are the appropriate initial antibiotics to order when concerned for the above uterine infection? (include dosage and frequency) __________________________________________ _____________________________________________________________________________ This regimen is the most effective. There are additional options; however, it is important to keep in mind that mothers are likely breastfeeding. ______________________ is an antibiotic contraindicated in breastfeeding.

Patients who are status post c-section or ill appearing will likely need to be admitted; consult Obstetrics and Gynecology (OB/GYN) for final disposition decisions and arrangement of close follow up if able to be discharged.

#### Section 3) Postpartum Cardio-Pulmonary Abnormalities

Given the significant changes in physiology that occur while pregnant, patients are at risk for the development of (choose one: dilated/restrictive) cardiomyopathy. This typically occurs in the ninth month of pregnancy or up to _____ months postpartum.

These are the risk factors for development of this disease: (list at least 5) _________________ _____________________________________________________________________________ But it can occur in anyone.

What are the likely presenting symptoms (historical features) and signs (exam features) of peripartum cardiomyopathy? List at least 3 of each Symptoms: ___________________________________________________________________ Signs: ________________________________________________________________________

These patients are managed as typical heart failure exacerbations because many of the classic treatment medications are acceptable postpartum with minimal impact during breastfeeding. How long after diagnosis, post-delivery, do you expect the patient to recover to baseline cardiac status? Will they recover? _________________________________________________ _____________________________________________________________________________

#### Section 4) Postpartum Disorders of Lactating Breasts

In the postpartum period, both mother and baby are acclimating to breastfeeding. It is not uncommon for women to present to the ER with breast pain or concern for an infection.

What is the condition which presents with pain and swelling of the breasts, possibly a low-grade fever, and on exam shows enlarged, hard breasts without erythema or focal swelling? ________________________ This condition typically presents _________ days postpartum. How will you counsel your patients to treat their symptoms? _________________________ ___________________________________________________________________________

What is the condition which presents with breast pain, erythema, warmth, and swelling? Pain is often severe, and patients will note fever, chills, and myalgias. ________________________ When evaluating this condition on point of care ultrasound, what might you expect to see? _____________________________________________________________________________

For the above infection, ______________ is the most common pathogen. How do women acquire this bacterial infection? ___________________________________________________

What is the treatment for this? (provide medication, dosage, frequency, length of treatment) _____________________________________________________________________________

How will you counsel your patients on managing their pain? ____________________________ _____________________________________________________________________________

In evaluating breast complaints in a lactating mother, though the above two conditions are most common, what other rare differential diagnosis must you consider? _________________ _____________________________________________________________________________

In a patient with recurrent mastitis or nipple pain, ask about breastfeeding habits and ease. It takes time and understanding of technique for patients and babies to successfully breastfeed. If your patients are having significant trouble, it could be related to their milk production.

In patients who underwent significant postpartum hemorrhage, ischemic injury to the pituitary gland can occur, limiting prolactin release and therefore a low or nonexistent milk supply. This condition is called _____________________________________________________________.

During pregnancy, patients with existing pituitary adenomas are also at risk for spontaneous hemorrhage or infarction called _____________________. This will often present with sudden onset severe headache, a concerning symptom in the intrapartum or postpartum period. However, both conditions may not present until later when endocrine dysfunction occurs.

### Postpartum Complications mTBL: Group Application Exercise (GAE)

#### Case 1

A 26 y/o female (F) presents to the ED with a fever. She is five days postpartum after a twin delivery complicated by an emergency cesarean section. She reports mild abdominal pain, increased lochia, and chills. She denies any respiratory symptoms. Vitals: Blood pressure (BP) 102/75, heart rate (HR) 110, temperature (T) 38.9° C, Oxygen Saturation (O2) 98% on room air (RA).

**What is your differential diagnosis?**
________________________________________________________________________
**What diagnostic studies are you ordering?**
________________________________________________________________________

#### Case 2

A 22 y/o F, Gravida(G)1Para(P)1, currently two weeks postpartum, presents to the ED complaining of dyspnea on exertion. She reports worsening leg swelling, mild central chest pain, and inability to walk across the room carrying her baby without stopping to catch her breath. She has significant orthopnea and has been sleeping in the recliner in her baby’s room. Her prenatal course was complicated by gestational hypertension which resolved at delivery, so she has no longer been taking her antihypertensive medications. Vital signs: BP 180/110, HR 115, RR 20, T 98.9° F, O2 95% on RA.

**What is your differential diagnosis?**
________________________________________________________________________
**What diagnostic studies are you ordering?**
________________________________________________________________________
**What is the treatment?**
________________________________________________________________________

#### Case 3

A 30 y/o F with asthma presents to the ED with breast pain. She is five weeks postpartum and has been struggling to breastfeed. Her child is gaining weight appropriately. On examination, she has a tender, erythematous left breast with central fluctuance. Vitals: BP 98/65, HR 102, T 37.9° C, O2 100% on RA.

**What is your differential diagnosis?**
________________________________________________________________________
**What diagnostic studies are you ordering?**
________________________________________________________________________ **A bedside ultrasound shows an anechoic fluid collection with overlying cobblestoning. What is your best next step**? ________________________________________________________________________

#### Case 4

A 33 y/o F presents to the ED complaining of headache. She is one week postpartum, and her delivery was complicated by hemorrhage requiring transfusion of 10 units of packed red blood cells (RBCs) with a prolonged post-op hospitalization. She is tearful when providing history, stating she presented directly from the pediatrician office and believes her headache is due to a migraine from stress because her child is not gaining weight. She feels inadequate as she is struggling to breastfeed. She denies any focal weakness, vision changes, leg swelling, nausea, vomiting, or other symptoms. She did not have any intrapartum complications.

**What is your differential diagnosis?**
________________________________________________________________________
**What diagnostic studies are you ordering?**
________________________________________________________________________

## INSTRUCTOR MATERIALS

Answer keys to all exercises with explanations, are on the following pages.

Learners: Please do not proceed.

### Postpartum Complications mTBL: Readiness Assessment Test Key (RAT Key)

#### Section 1) General Postpartum Information

Complications unique to the postpartum period typically begin about 48-hours after delivery. For how long after the 48-hour period are patients at increased risk of postpartum complications?

***6 weeks***

List the possible postpartum complications patients may present to the emergency department with: ***Hypertension, hemorrhage, pre-eclampsia/eclampsia, infection (endometritis), cardiomyopathy, depression or psychosis, mastitis or engorgement, Sheehan syndrome/pituitary apoplexy, cerebral venous thrombosis, and many more.***

We commonly think about postpartum hemorrhage immediately following delivery, with the most common cause being uterine atony. However, patients can present with **late** postpartum hemorrhage, even weeks following delivery. What is the differential diagnosis for late postpartum hemorrhage? ***Undiagnosed coagulopathy, endometritis, retained placental products, delayed placental site involution, non-healing laceration, retained foreign bodies (lap pads).***

A systolic BP > ***140*** and a diastolic BP > ***90*** are concerning for a significant intrapartum or postpartum abnormality. Elevated blood pressure should cause you to consider ***preeclampsia/HELLP*** (**H**emolysis, **E**levated **L**iver enzymes and **L**ow **P**latelets) which can develop acutely in the postpartum period. Pay careful attention to the BP of all young women in the event they are recently postpartum and did not say such in their initial triage assessment.

It is important to be aware of postpartum hemorrhage and postpartum preeclampsia as key contributors to morbidity and mortality in our pregnant patients. These topics are vast and need their own dedicated consideration, so we will not be covering them further today.

#### Section 2) Postpartum Infections

As with any procedure or surgery, pregnant patients are at risk for infections following delivery.

***Endometritis*** is the name of the uterine infection occurring postpartum, and you should assume this diagnosis, until proven otherwise, in any postpartum patient with a fever.

What are the risk factors for acquiring this uterine infection? ***C-section, multiple gestation, younger age, prolonged labor, prolonged rupture of membranes, internal fetal monitoring, lower socioeconomic level, maternal human immunodeficiency virus (HIV), and any digital cervical examination after 37 weeks’ gestation (hence need for sterile gloves for cervical exams)***. Of these risk factors, ***c-section*** is the most significant risk factor.

What are the most common bacteria causing uterine infections in the postpartum period? ***Polymicrobial: Gram positive (Staphylococcus and Streptococcus), gram negative (Escherichia Coli), anaerobes (peptostreptococcus), and sexually transmitted infections (STI) Group B Streptococcus, chlamydia, gonorrhea, mycoplasma.***

What are the appropriate initial antibiotics to order when concerned for the above uterine infection? (include dosage and frequency) ***Clindamycin + Gentamicin (or another aminoglycoside). Dose= Clindamycin 900 mg q8h + Gentamicin 5mg/kg q24 hours.*** This regimen is the most effective. There are additional options; however, it is important to keep in mind that mothers are likely breastfeeding. ***Doxycycline*** is an antibiotic contraindicated in breastfeeding.

Patients who are status post c-section or ill appearing will likely need to be admitted; consult OB/GYN for final disposition decisions and arrangement of close follow up if able to be discharged.

#### Section 3) Postpartum Cardio-Pulmonary Abnormalities

Given the significant changes in physiology that occur while pregnant, patients are at risk for the development of (choose one: ***dilated/***restrictive) cardiomyopathy. This typically occurs in the ninth month of pregnancy or up to ***5 months*** postpartum.

These are the risk factors for development of this disease: (list at least 5) ***cardiac disease in prior pregnancies, multiple gestations, older maternal age, black race, chronic hypertension or gestational hypertension, intrapartum complications with preeclampsia and eclampsia or HELLP.*** But it can occur in anyone.

What are the likely presenting symptoms (historical features) and signs (exam features) of peripartum cardiomyopathy? List at least 3 of each

Symptoms: ***dyspnea, orthopnea, cough, palpitations, chest pain, edema*** Signs: ***pulmonary crackles, jugular venous distension (JVD), increased work of breathing, low oxygen saturation, hypertension, edema***

These patients are managed as typical heart failure exacerbations since many of the classic treatment medications are acceptable postpartum with minimal impact during breastfeeding. How long after diagnosis, post-delivery, do you expect the patient to recover to baseline cardiac status? Will they recover? ***Recovery to normal ejection fraction (EF) is typically seen after 6 months and occurs in most women. However, they are prone to dysrhythmias and may need implantable pacemakers. They are also at increased risk for the development of cardiomyopathy in future pregnancies.***

#### Section 4) Postpartum Disorders of Lactating Breasts

In the postpartum period, both mother and baby are acclimating to breastfeeding. It is not uncommon for women to present to the ER with breast pain or concern for an infection.

What is the condition which presents with pain and swelling of the breasts, possibly a low-grade fever, and on exam shows enlarged, hard breasts without erythema or focal swelling? ***Engorgement***. This condition typically presents ***3–5 days*** postpartum. How will you counsel your patient to treat their symptoms? ***Frequent pumping or feeds to help release milk adequately, compresses, acetaminophen or non-steroidal anti-inflammatory drugs (NSAIDs) for pain.***

What is the condition which presents with breast pain, erythema, warmth, and swelling? Pain is often severe, and patients will note fever, chills, and myalgias. ***Mastitis.*** When evaluating this condition on point of care ultrasound, what might you expect to see? ***Hypoechoic fluid in fat globules, causing cobblestoning appearance, without a discrete pocket to suggest an abscess.***

For the above infection, ***Staphylococcus Aureus*** is the most common pathogen. How do women acquire this bacterial infection? ***Retrograde passage of skin flora into microscopic abrasions on the breast from the baby’s mouth. General nipple irritation can be caused by poor latching techniques.***

What is the treatment for this? (provide medication, dosage, frequency, length of treatment) ***Continue breastfeeding, pain control with acetaminophen or NSAIDs, dicloxacillin (500mg QID x10 days) or cephalexin 500 mg QID x10 days). As mentioned above, doxycycline and metronidazole are contraindicated in breastfeeding.***

How will you counsel your patients on managing their pain? ***Cold compresses to ease swelling, medication management with acetaminophen or NSAIDs.***

In evaluating breast complaints in a lactating mother, though the above two conditions are most common, what other rare differential diagnosis must you consider? ***Inflammatory breast cancer.***

In a patient with recurrent mastitis or nipple pain, ask about breastfeeding habits and ease. It takes time and understanding of technique for patients and babies to successfully breastfeed. If your patients are having significant trouble, it could be related to their milk production.

In patients who underwent significant postpartum hemorrhage, ischemic injury to the pituitary gland can occur, limiting prolactin release and therefore a low or nonexistent milk supply. This condition is called ***Sheehan syndrome.***

During pregnancy, patients with existing pituitary adenomas are also at risk for spontaneous hemorrhage or infarction called ***pituitary apoplexy***. This will often present with sudden onset severe headache, a concerning symptom in the intrapartum or postpartum period. However, both conditions may not present until later when endocrine dysfunction occurs.

### Postpartum Complications mTBL: Group Application Exercise (GAE) Key

#### Case 1

A 26 y/o female (F) presents to the ED with a fever. She is five days postpartum after a twin delivery complicated by an emergency cesarean section. She reports mild abdominal pain, increased lochia, and chills. She denies any respiratory symptoms. Vitals: Blood pressure (BP) 102/75, heart rate (HR) 110, temperature (T) 38.9° C, Oxygen Saturation (O2) 98% on room air (RA).

**What is your differential diagnosis?** *Endometritis, urinary tract infection (UTI)/pyelonephritis, cellulitis or dehiscence of c-section scar, retained placental products, septic thrombophlebitis, mastitis, necrotizing fasciitis.* **What diagnostic studies are you ordering?** *Complete blood count (CBC), basic metabolic panel (BMP), urine analysis (UA), blood cultures, computed tomography scan (CT) of the abdomen (evaluate for endometritis, retained products, deeper abscesses). If learners mention chest x-ray (CXR), this would be warranted if the patient had respiratory symptoms.* **Facilitator guide:** As with all patients presenting to the ED, it is paramount to keep a broad differential diagnosis and consider all possible sources of sepsis in this patient. It is also important to ensure a thorough exam is performed to evaluate all wounds for infection because life-threatening disorders such as necrotizing fasciitis are a concern and must be ruled out. These patients should be treated as septic with early initiation of fluids and intravenous (IV) antibiotics. Transvaginal ultrasound (TVUS) can also evaluate for retained products of conception; however, CT is preferred in this case given possible abscess secondary to surgical procedure.

#### Case 2

A 22 y/o F, Gravida(G)1Para(P)1, currently two weeks postpartum, presents to the ED complaining of dyspnea on exertion. She reports worsening leg swelling, mild central chest pain, and inability to walk across the room carrying her baby without stopping to catch her breath. She has significant orthopnea and has been sleeping in the recliner in her baby’s room. Her prenatal course was complicated by gestational hypertension which resolved at delivery, so she has no longer been taking her antihypertensive medications. Vital signs: BP 180/110, HR 115, RR 20, T 98.9° F, O2 95% on RA.

**What is your differential diagnosis?** *Postpartum cardiomyopathy, pre-eclampsia, acute coronary syndrome, pulmonary infection, pulmonary embolism.* **What diagnostic studies are you ordering?** *Electrocardiogram* (*ECG), troponin, brain natriuretic peptide (BNP), CXR, and bedside echocardiogram; if high suspicion for pulmonary embolism (PE) can do CT angiography (CTA); d-dimer would be reasonable here since the diagnosis is more likely cardiomyopathy.* **What is the treatment?** *Nitroglycerin, positive pressure ventilation as needed, furosemide as needed, and if severe, mother may need nitro drip. Treatment is like congestive heart failure (CHF) exacerbation, oxygen as needed, positive pressure ventilation, diuretics, and if severe, vasodilators and arrhythmia management. Learners should also empirically give magnesium since she remains somewhat undifferentiated, and we want to prevent seizures/progression to eclampsia.* **Case 2 Facilitator guide:** Postpartum cardiomyopathy is more common in older patients with multiple gestations, but it can present in anyone. It is often associated with superimposed preeclampsia, obesity, or coexisting infection. Patients present with evidence of left heart failure: fluid overload manifested as pulmonary edema, jugular venous distension, dyspnea on exertion, and orthopnea. Treatment is like CHF, oxygen as needed, positive pressure ventilation, diuretics, and if severe, vasodilators and arrhythmia management.

#### Case 3

A 30 y/o F with asthma presents to the ED with breast pain. She is five weeks postpartum and has been struggling to breastfeed. Her child is gaining weight appropriately. On examination, she has a tender, erythematous left breast with central fluctuance. Vitals: BP 98/65, HR 102, T 37.9° C, O2 100% on RA.

**What is your differential diagnosis?** *Engorgement, mastitis, breast abscess, inflammatory breast cancer, hidradenitis suppurativa.* **What diagnostic studies are you ordering?** Point of care ultrasound (*POCUS) +/− formal ultrasound; no lab work is necessary here to diagnose condition. If you do order: CBC, BMP, lactic acid. May need surgical consult.* **A bedside ultrasound shows an anechoic fluid collection with overlying cobblestoning. What is your best next step**? *Consult surgery for fine needle aspiration.* **Case 3 Facilitator guide**: Not all breast pain and fever are mastitis; we must also consider engorgement which would not indicate treatment with antibiotics. Rarely, inflammatory breast cancer can present in this period and may require surgical biopsy. The most common infection will be mastitis. Breast abscesses are overall rare complications of mastitis, occurring in only about three percent of patients and typically after first failing a course of antibiotics. However, we must consider them in cases where mothers fail to respond to outpatient antibiotics. Ultrasound guided aspiration is a preferred initial method of drainage to incision and drainage (I&D) because surgical incisions can be complicated by the development of milk fistulas. Treatment is with cefalexin or clindamycin; if septic, can consider IV vancomycin. Bactrim is contraindicated in breastfeeding infants less than two months of age. Mothers should be encouraged to continue breastfeeding through any infections, if the antibiotic choice is appropriate.

#### Case 4

A 33 y/o F presents to the ED complaining of headache. She is one week postpartum, and her delivery was complicated by hemorrhage requiring transfusion of 10 units of packed red blood cells (RBCs) with a prolonged post-op hospitalization. She is tearful when providing history, stating she presented directly from the pediatrician office and believes her headache is due to a migraine from stress because her child is not gaining weight. She feels inadequate as she is struggling to breastfeed. She denies any focal weakness, vision changes, leg swelling, nausea, vomiting, or other symptoms. She did not have any intrapartum complications.

**What is your differential diagnosis?** *Sheehan syndrome, pituitary apoplexy, cerebral venous thrombosis, intracranial hemorrhage, post dural puncture headache, preeclampsia, tension or migraine headache.* **What diagnostic studies are you ordering?** *None; this is a clinical diagnosis. Outpatient magnetic resonance imaging (MRI) and prolactin levels can be obtained. Infant should be treated with formula supplementation.* **Case 4 Facilitator guide**: Headaches are common in the postpartum period, with most of them of benign etiology such as tension headaches. In this case, the headache is a distractor, and the patient has Sheehan syndrome, a complication of severe postpartum hemorrhage resulting in ischemia of the pituitary gland. This often presents as difficulty breastfeeding. Take care to obtain clear history and avoid assuming a pregnant patient’s headache is secondary to stress. The learners should have considered a full differential diagnosis of postpartum headache even if they realized this was Sheehan syndrome. Post dural puncture headaches are common and may require blood patching. These typically present with a headache which is worse when in upright position and improved with lying flat. It is important to consider life-threatening causes such as cerebral venous thrombosis, intracerebral hemorrhage, and pre-eclampsia. These may or may not have neurologic symptoms, and patients should be assessed for onset of the symptoms and will require advanced imaging if any are highly suspected. To diagnose central venous sinus thrombosis, MRI and magnetic resonance venogram (MRV) are gold standard. Pre-eclampsia is diagnosed with hypertension plus evidence of end organ damage. The definition has recently been expanded to include headache that does not respond to acetaminophen as evidence of end organ damage. This patient’s vitals are stable and reassure against pre-eclampsia.
